# Evaluation and Improvement of the Current CRCP Pavement Design Method

**DOI:** 10.3390/ma16010358

**Published:** 2022-12-30

**Authors:** Milad Moharekpour, Pengfei Liu, Markus Oeser

**Affiliations:** 1 Institute of Highway Engineering, RWTH Aachen University, Mies-van-der-Rohe-Str. 1, 52074 Aachen, Germany; 2 Federal Highway Research Institute (BASt), Brüderstr. 53, 51427 Bergisch Gladbach, Germany

**Keywords:** CRCP, concrete pavement, crack width, crack spacing, AASHTO, MEPDG

## Abstract

Continuously reinforced concrete pavement (CRCP) is a representative type of concrete pavement constructed with continuous steel bars without intermediate transverse expansions. With reference to pavement conditions, CRCP is an exceptional type of concrete pavement according to the Highway Pavement Condition Index (HPCI) and International Roughness Index (IRI). The two main design methods for CRCP are AASHTO 86/93 and the Mechanistic–Empirical Pavement Design Guide (MEPDG). Because of limitations of the AASHTO 86/93 design method, the MEPDG method is more reliable. While incorporating the interactions among geometrics, pavement structure layers, material properties, subgrade, traffic, and environmental conditions, and the prediction values according to the MEPDG method, it matched the measured results of crack spacing and crack width. The MEPDG punchout, crack width and spacing, and load transfer efficiency (LTE) models were evaluated, and results were compared with the test sections in three European countries consisting of different construction details, which were investigated and recorded between 2019 and 2021. In this sense, a calculation tool was developed to consider the influence of different parameters in design process. In addition, sensitivity analyses were executed for the development of punchout, considering various input parameters. The track surveying and the evaluation of the results indicated that the design process can be improved with consideration of some criteria such as crack formation time or adjustment of the correlation between crack width and crack spacing. Due to the very important function of erosion and resulting pumping in the deterioration of CRCP, it is advisable to include the influence of the base layer and the influence of different shoulder type and heavy traffic volume or effect of deflection in the calculations.

## 1. Introduction

**Figure 1:**
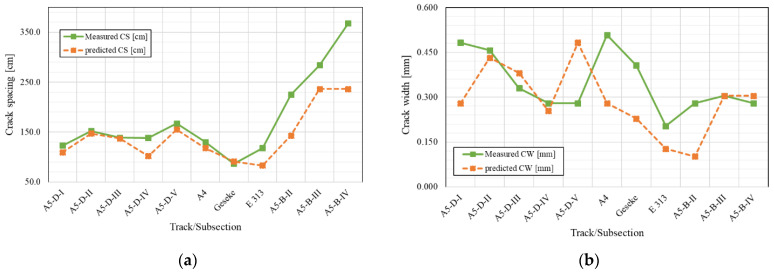
Predicted and measured values for considered tracks/subsections: (**a**) crack spacing [CS]; (**b**) crack width [CW].

Continuously reinforced concrete pavement (CRCP) is a characteristic type of concrete pavement constructed with continuous steel bars without intermediate transverse expansions. In terms of pavement conditions, CRCP is a superior type of concrete pavement according to the Highway Pavement Condition Index (HPCI) and International Roughness Index (IRI). The steel reinforcement manages the occurrence of random transverse cracks resulting from volumetric changes due to cement–water hydration and holds the cracks tightly together such that the concrete slab behaves like a continuous system to transfer loads across the crack interface [1,2,3]. The lack of transverse joints provides CRCP with long life performance with minimal maintenance and sustained smoothness, and it can be an excellent pavement approach to accommodate heavily loaded traffic because of the high yield and tensile strength characteristics [4,5,6]. The performance of CRCP is related to multiple design, material, and construction parameters and is greatly affected by two limiting criteria: crack spacing and crack width. It is also affected by reinforcement ratio, slab thickness, traffic load, concrete cover of reinforcement, and base type [7,8]. The two main design methods for CRCP are the American Association of State Highway and Transportation Officials (AASHTO) 86/93 and the Mechanistic–Empirical Pavement Design Guide (MEPDG) developed under NCHRP I-37A. The AASHTO 86/93 guide limits the crack spacing between 0.91 m and 1.83 m to reduce CRCP distresses [9]. To minimize the potential for punchout, the minimum preferable crack spacing to be designed is 1.07 m, and, to reduce the incidence of spalling, the maximum spacing between consecutive cracks should be no more than 2.44 m [10]. Because of limitations of the AASHTO 86/93 design method, the MEPDG method is more reliable, which incorporates the interactions among geometrics, pavement structure layers, properties of materials, subgrade, traffic, and environmental conditions. The prediction values depending on the MEPDG method matched the measured results of crack spacing and crack width recorded on the test sections in European countries with different construction details more closely [11,12,13]. The key performance indicators in the MEPDG design procedure, based on the incremental approach of the design process, are the quantity of punchouts and roughness measured with the International Roughness Index (IRI). However, values for the other known performance indicators such as crack spacing, crack width, and load transfer efficiency are also determined as part of the calculations, as they also contribute significantly to the extent of the damage. The method calculates the degree of deterioration of the concrete pavement by estimating the degradation of various functional properties, and the results are presented incrementally over the service life [14]. The major structural distress types for CRCP are punchouts, wide transverse cracks, and longitudinal cracks [15]. An analysis of 47 long-term pavement performance (LTPP) sections showed that the possibility of punchouts develop on the CRCP when the average crack spacing is between 0.3 to 0.6 m [16]. The development of punchout is dominated by the maximum tensile stress at the top of the concrete slab [15]. The other performance factor for CRCP is crack width, which ensures an adequate load transfer efficiency (LTE) of cracks, as well as minimizes water infiltration into the pavement and the penetration of incompressible materials [17]. The AASHTO Interim MEPDG forecasts and recommends a maximum crack width of 0.5 mm at the reinforcement depth over the entire design life to maintain crack LTE at high levels and minimize possible corrosion of the steel [18]. A comparison of crack spacing and crack width calculated by MEPDG against experimental CRCP sections showed that prediction values did not exactly match the measured punchouts, crack spacing, and crack width [19]. By re-dimensioning the 13 tracks/sections with different technical data and service life, it was found that MEPDG design method is more effective than the AASHTO method in predicting crack width and spacing. Despite better forecast, there are still some inconsistencies between MEPDG values and measured values. Figure 1 represents the predicted and measured crack spacing and crack width for considered tracks.

The reason for these discrepancies between predicted MEPDG values and measured values is the deficiencies in the design method, which have important functions in the design of CRCP and are not considered in the procedure of MEPDG [11].

**Figure 2:**
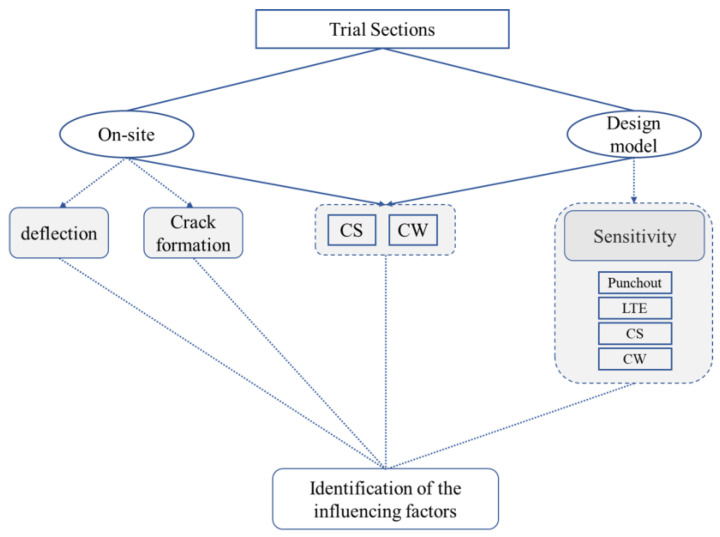
Methodology to identify the influencing factors that are not embedded in the design procedure [CS: crack spacing; CW: crack width].

The objective of this publication (Figure 2) is to identify the influencing factors that are not embedded in the design procedure of MEPDG based on the existing long-term experiences from test tracks. This was achieved through performing a sensitivity analysis for different variables of punchout model to determinate performance indicators such as crack spacing, crack width, and LTE, as well as the evaluation and interpret of the on-site tests.

## 2. Design Logic in MEPDG

Although MEPDG is an incremental approach to the design process, and hundreds of thousands of stress calculations are required to estimate damages on a monthly basis over a design period of many years, material parameters of concrete (the modulus of elasticity E_PCC,i_, modulus of rupture MR_PCC,i_, tensile strength f_t,i_, and compressive strength f_c,i_) are calculated monthly, whereas traffic load and ambient temperature are calculated up to hourly. The drying shrinkage has a significant influence on the crack characteristics that develop at an early age and depend on the humidity in the concrete. Another important component for the CRCP calculations is the drop in *PCC* temperature at the depth of steel Δ*T*_ζ,*m*_. The modulus of elasticity of the subbase (*E_SB_*) and the modulus of subgrade reaction (*k*) are also important in the MEPDG design method. The last time-dependent parameter to be determined is the radius of relative stiffness *l_i_* This value is used to describe the relative stiffness of the concrete slab compared to its foundation. The crack spacing is dependent on CRCP age, traffic load, and fatigue, and its calculation in the MEPDG includes the tensile strength of the concrete after 28 days f_t_ (psi), the Bradbury’s curling/warping stress coefficient *C*, Westergaard’s nominal stress factor *σ*_0_ (psi), the depth to steel layer *ζ* (in), the slab thickness *h_PCC_* (in), the coefficient of base friction *f*, the peak bond stress *U_m_* (psi), the steel ratio *P_b_*, the coefficient of first bond stress *c*_1_, and the reinforcing steel bar diameter *d_b_* (in). At an early age, temperature and moisture variations induce volume changes in the concrete, which are arrested by the reinforcement and the base friction, leading to the development of stresses and occurrence of transverse cracks, because the tensile stress exceeds the concrete’s strength [1,20].

The average crack width coming from MEPDG is calculated as follows [18]:

(1) $$cw_{i}=CC\times L\times \left(\varepsilon_{tot}-\frac{c_{2,i}\times f_{\sigma ,i}}{E_{PCC,i}}\right)\times 1000,$$

(2) $$\varepsilon_{tot}=\varepsilon_{shr,i}+\alpha_{PCC}\times \Delta T_{\zeta ,m},$$

where *CC* is the calibration constant (according to the global calibration, it is recommended in MEPDG to be 1), L is the mean crack spacing, *ε_shr,I_* is the strain at the depth of the steel from shrinkage [10^−6^ in/in], *α_PCC_* is the *PCC* coefficient of thermal expansion [°F^−1^], Δ*T_ζ,m_* is the drop in *PCC* temperature from zero-stress temperature at the depth of the steel at the time of crack width prediction [°F], *c*_2_ is the coefficient of second bond stress, depending on concrete strength and crack spacing by 0 °C at the level of reinforcement and zero temperature differential, *EPCC* is the concrete elastic modulus, and *f_σ_* is the maximum longitudinal tensile stress in the concrete at the level of reinforcement. The above equation provides the crack width according to total strain (from shrinkage and temperature drop) at the level of steel and the restrained longitudinal deformation caused by stresses in the concrete.

For CRCP, major structural distresses considered for design are punchouts, which are a result of water infiltration due to wider crack opening in combination with erosion of the base layer due to pumping effects, resulting in a CRCP pavement which is no longer completely supported; therefore, additional stresses appear in the concrete with closer transverse cracks and longitudinal cracks occurring [10,21,22]. The number of medium- and high-severity punchouts can be calculated using the following equation:

(3) $$PO_{i}=\frac{A}{1+\alpha \;\cdot \;FD_{PO}^{\beta }},$$

where *PO_i_* is the total predicted number of punchouts at the end of the time increment, *FD_PO_* is the accumulated fatigue damage, and *A*, *α*, and *β* are the calibration constants for the punchout function. The maximum top of concrete transverse tensile stress is calculated depending on the traffic level and axle load distributions for each time increment. Damage is calculated on the basis of the stress level and traffic load applications. This damage is accumulated over time, and the possibility of punchout is calculated.

## 3. Pavement Structures

Between 2019 and 2021, sections in Germany, Belgium, and Poland were investigated in the field, along with the help of extracted drill cores in the laboratory (Table 1). A5 Bruchsal and A5 Darmstadt are abbreviated to A5 B and A5 D, respectively.

**Figure 3:**
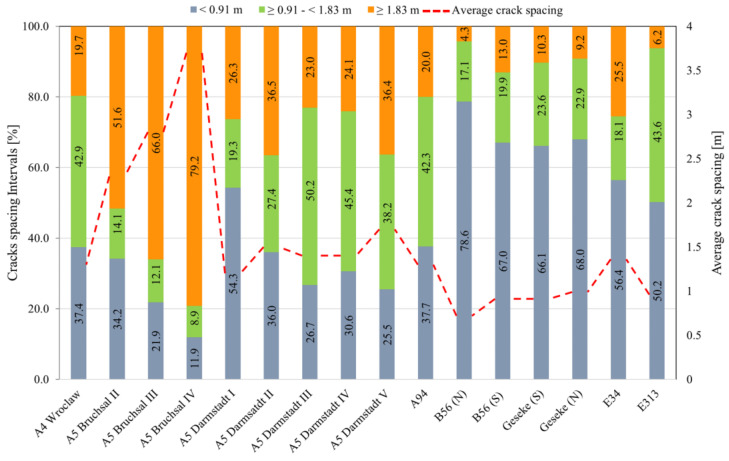
Classification of the crack spacing of the studied sections.

Figure 3 summarizes the categorization of crack spacing into three classes: less than 0.91 m, between 0.91 m and 1.83 m, and greater than 1.83 m. It also shows the average crack spacing of each section.

The time of crack assessment and evaluation of the crack spacing are presented in Table 1 by service life. In two sections (A5 Bruchsal II und A5 Bruchsal III), crack surveys were performed 1 year after concrete placement. Crack development was likely not completed at that time and the A5 Bruchsal IV was covered with a thin overlay (1.5 cm) and only the reflected crack was recorded. Twelve of the 16 sections surveyed had average transverse crack spacings within the desired range of 0.91 m to 1.83 m. Regarding the crack width, only the information from 11 tracks/subsections is available; for example, A5 Bruchsal and A94 were overlaid shortly after concrete placement. In addition, E40 was overlaid in 2007, and, since the cracking was completed, the cracks have not been reflected.

## 4. Results and Analysis

### 4.1. Crack Formation

**Figure 4:**
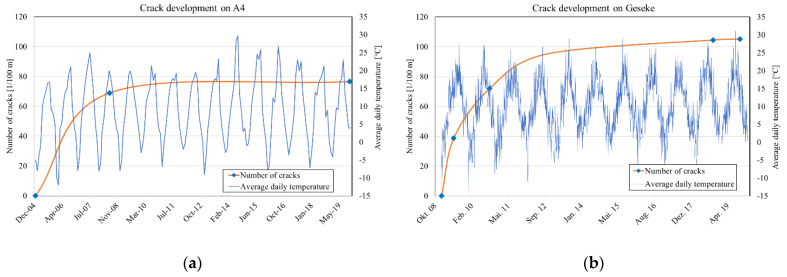
Development of the crack pattern from placement to 2019: (**a**) A4 (PL), (**b**) Geseke (DE).

Equation (1) assumes that the cracks occur directly after the concrete is placed and no more cracks occur over the course of time, although the formation of cracks is completed after 2–4 winter periods, depending on the climatic conditions [1,2]. The development of cracks per 100 m is shown for the A4 and Geseke tracks as a function of the daily mean temperature (Figure 4). In both tracks, the completion of the crack formation is represented on the basis of winter periods.

**Figure 5:**
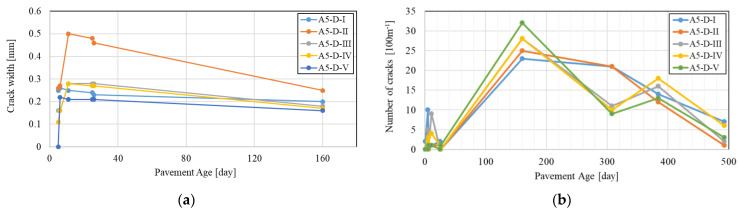
Formation of cracks on five different sections of A5 near Darmstadt: (**a**) development of crack width; (**b**) number of cracks per 100 m.

Moreover, the cracks that form at early ages have a larger opening width compared to the cracks that occur later and after the completion of the drying shrinkage process. Figure 5a shows the development of the crack width in five sections of A5 near Darmstadt within 160 days after placement, representing only the newly developing cracks during respective roadway surveys. The track surveying indicated that the opening widths of the cracks are contracting, although, in the calculations according to MEPDG, the average crack width increases over time due to the continued drying shrinkage of concrete. It can be assumed that the crack width near the depth of longitudinal steel also decreases because the potential of volume change in the reinforcement layer is smaller than the concrete surface due to higher relative humidity and lower temperature changes. Equation (1) implies that concrete volume changes contributing to crack widths at the steel depth encompass all concrete between the two transverse cracks, although the concrete in slab with a certain distance to the transverse crack has no movements that affects the crack width. However, field tests performed as part of TxDOT’s 0–1700 research study indicated that the limitation of the volume change of the concrete due to the longitudinal steel is limited to a distance of about 30 cm [23] from the transverse cracks, although this distance may vary depending on the environmental conditions.

**Figure 6:**
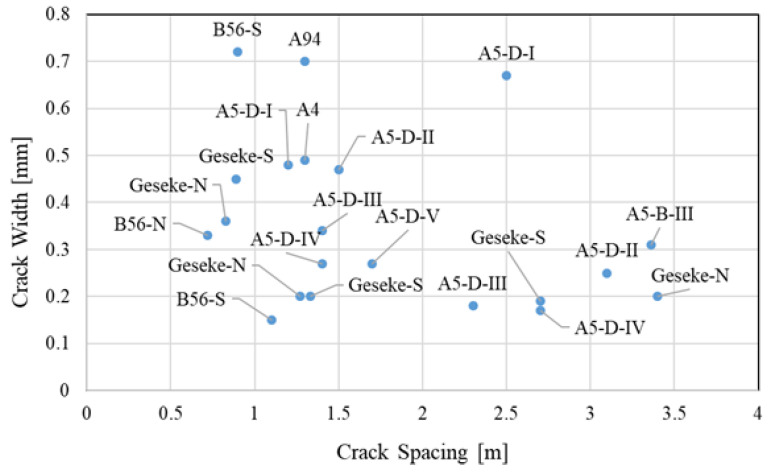
Correlation between crack spacing and crack width.

Figure 5b shows the crack initiation on the A5 near Darmstadt, which was recorded 10 times in 493 days after concrete placement. Only the newly formed cracks are shown in the diagram. It indicates that transverse cracks develop early after paving, but continue to form and stabilize over time. Incidentally, Equation (1) assumes that there is a linear correlation between the crack spacing and crack width, where increasing the crack spacing increases crack width, which increases the LTE. In contrast, field studies indicated that there is almost no correlation between crack spacing and crack width measured on the concrete surface, as shown in Figure 6.

The crack pattern of CRCP, characterized by crack distances and crack widths, significantly determines the performance of this type of pavement in the short and long term. Cracks too close to each other can, in combination with other detrimental traffic load factors, water infiltration, or poor-quality foundations, give rise to the punchout damage phenomenon. Cracks that are too wide result in a larger crack width and have a higher risk that water penetrates the structure causing corrosion of the reinforcing steel and pumping effects, which may also result in punchout and horizontal crack formation. Therefore, it is possible to guide the crack pattern by inserting saw cuts over limited length at the side of the concrete slab to come to a more optimized crack distribution. The crack survey on the E313 in 2020 provided very positive results with regard to crack initiation. Approximately 8 years after concrete placement, 76% of the cracks were within a targeted crack spacing area, while 85% of the notches (60 mm depth, 40 cm length at a distance of 120 cm) were active. Despite very successful experience with the crack controlling system and its influence on obtaining a more homogeneous crack distribution, the application of the crack initiation technique was not integrated in the design method.

### 4.2. Crack Width

**Figure 7:**
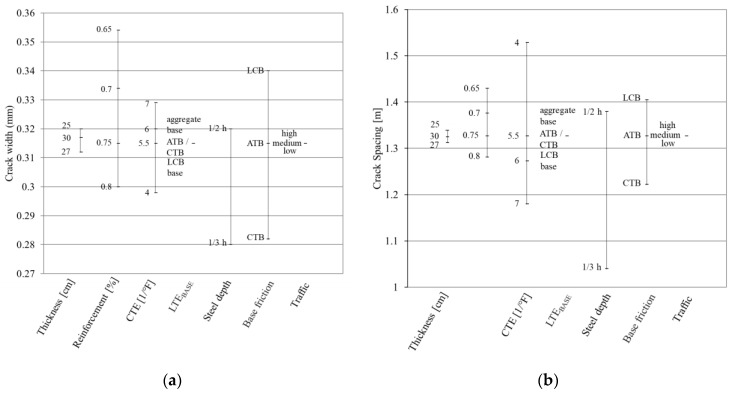
Relative impact of the individual variables on the (**a**) crack spacing, and (**b**) crack width by the MEPDG. ATB: asphalt-treated base; CTB: cement-treated base, LCB: lean concrete base.

The most important element that influences punchout development in MEPDG is crack width which is affected by crack spacing because of its major impact on crack width calculation. With respect to the lack of correlation between crack width and crack spacing and their impression on the design model, the punchout prediction is affected. Furthermore, crack stiffness, which is a function of crack width, is an essential component to LTE calculation. The sensitivity of the predicted crack spacing and crack width to each of the input parameters applied in the design method is illustrated in Figure 7. Accordingly, different values for each input parameter were investigated in order to find out the sensitivity of the method depending on different scenarios. For example, four different reinforcement ratios were individually imported into the design algorithm, and the resulting crack width and crack spacing were calculated.

It can be observed that the calculated crack width and crack spacing are responsive to the same parameters; with an increase in the reinforcement ratio, the coefficient of thermal expansion (CTE), steel covering, and friction type, the crack spacing and crack width decrease. Crack width is highly dependent on the reinforcement ratio and base friction, whereas crack spacing is strongly influenced by the CTE and steel depth. Both criteria are insensitive to volume of traffic, thickness, and LTE of the base. It is advised to keep the reinforcement rate as exact as possible to decrease the crack spacing and crack width. In this way, the crack activity due to temperature variations will be limited, and the reflection of the crack through the asphalt overlay will be avoided. To keep the CRCP economic, both a minimum layer thickness and a maximum layer thickness must be calculated so that the degree of reinforcement, which depends on the thickness, is not strongly influenced. An increase in the thickness from 25 cm to 27 cm would result in a decrease in reinforcement rate from 0.75% to 0.70%. This would have consequences in the crack spacing and crack width. In addition, an increase in the thickness of the concrete pavement would result in a decrease in the bending stresses due to traffic load at the bottom of the concrete slab. This would, therefore, result in a larger crack spacing, with a more opening crack width as the reinforcement rate is lower.

### 4.3. Punchout

**Figure 8:**
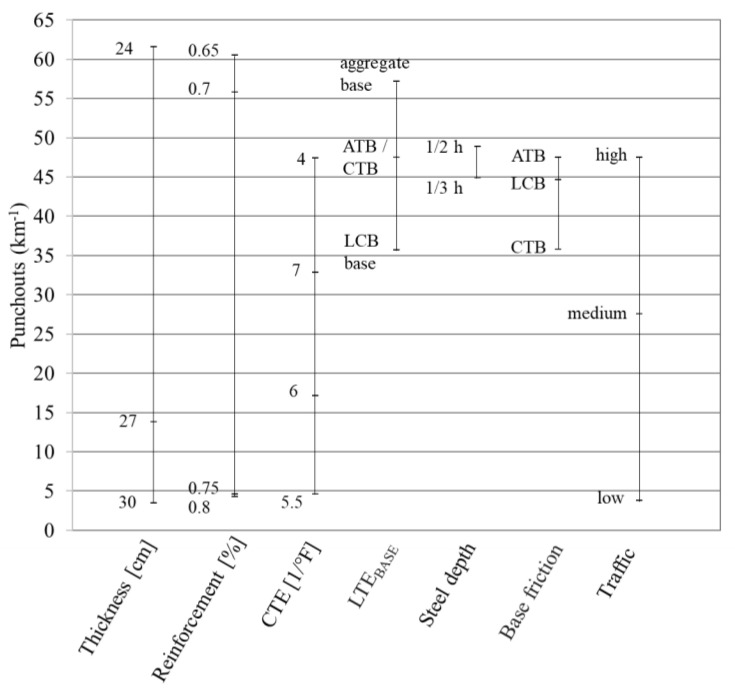
Relative impact of the individual variables on the punchouts prediction by the MEPDG.

According to the MEPDG, crack widths and the resulting loss of LTE in transverse cracks are the main causes of punchout [24]. The sensitivity of the predicted punchout to each variable characterized by the MEPDG at the end of the 40th design life is summarized in Figure 8. According to Figure 8, the punchout model is highly sensitive to slab thickness, reinforcement ratio, CTE, and volume of traffic, and moderately sensitive to base type and base friction, although punchouts in CRCP are mainly caused by localized poor support of the slab.

**Figure 9:**
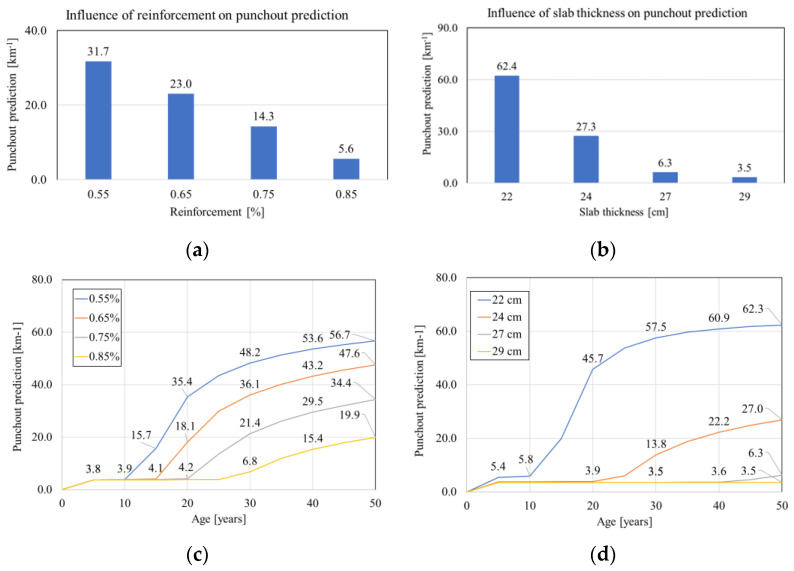
Sensitivity of punchouts to (**a**) reinforcement, (**b**) slab thickness, (**c**) reinforcement/age, and (**d**) slab thickness/age.

Figure 9 indicates the influence of different reinforcement ratios and slab thicknesses on punchout prediction. According to the MEPDG, the influence of slab thickness is quite substantial and much higher than that of the reinforcement ratio. Increasing the layer thickness from 22 cm to 29 cm decreased the punchout probability from 62.4 to 3.5 per km (Figure 9b).

This difference of influence is due to the direct involvement of the slab thickness in the calculations of loss in shear capacity (Δs_i_), although, in these calculations, the reinforcement ratio is contributed to indirectly by the use of the crack width. However, the evaluation of the recorded parameters on the test tracks is not in agreement with the design procedures prescribed in the MEPDG, which provided different results. On the Geseke, with an in situ layer thickness of 22.6 cm and a reinforcement ratio of 0.79%, 105 cracks per 100 m were recorded after 10 years, and the FWD values showed the failure of the bearing capacity. On section II of the A5 near Darmstadt with a layer thickness of 28.2 cm and a reinforcement ratio of 0.64%, after only 4 years, 197 cracks per 100 m were recorded, and the average crack spacing was 0.5 m. According to the MEPDG for the given input data for the E313 track, under consideration of the current construction age (1972), 34.3 punchouts per km were expected, although no punchouts were recorded after the current track inspection.

Although the development of punchouts is affected by erosion and loss of support of subgrade due to base erosion, the influence of subgrade is not considered in the MEPDG design method, and cracking from top to bottom is only included in the punchout procedure [8,25]. From another perspective, the effect of reinforcement cover and month of construction on the development of punchouts is not directly included in the design calculations, although this impact has already been confirmed.

The analysis of the IRI and punchout development according to the MEPDG on A5 Darmstadt consisting of five different sections with uniform concrete thickness and reinforcement ratio and various base types clearly showed the high influence of base layers. However, the FWD measurements on the track 15 years after the construction gave slightly different values regarding the load-bearing capacity of the sections.

Despite uniform concrete thicknesses and reinforcement degrees, different FWD values were observed. Although the use of geotextile reduced the load-bearing capacity (sections II and III), uniform crack spacing and narrower cracks were recorded in these sections.

The majority of damage to CRCP in the form of punchouts is caused by quality problems in the materials or substandard construction practice near the joint and the repair joint, and this issue is not involved in the design method, although, in the calculations of the IRI, the condition of the pavement in the form of initial as-constructed IRI, as well as the site factor, is included in the algorithm:

(4) $$IRI=IRI_{i}+C_{1}\cdot \;PO+C_{2}\cdot \;SF,$$

where *IRI_i_* is the initial *IRI* [in/mi], *C*_1_ and *C*_2_ are constant factors, *PO* is the number of punchouts [1/mi], and *SF* is the site factor.

### 4.4. Load Transfer Efficiency

**Figure 10:**
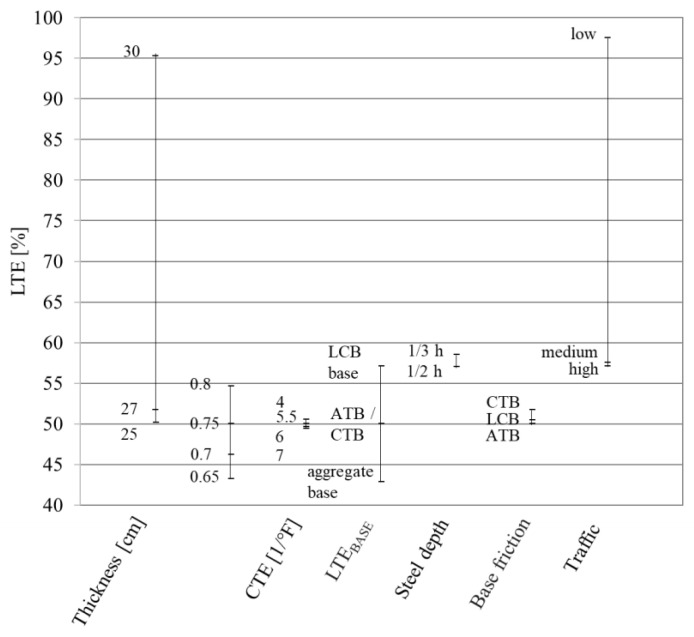
Relative impact of the individual variables on the LTE prediction by the MEPDG.

As indicated in Figure 10, the LTE of transverse cracks is highly sensitive to both slab thickness and the volume of traffic

Conversely, the LTE is only moderately sensitive to reinforcement ratio and base type, while it is insensitive to the coefficient of thermal expansion (CTE), steel depth, and base friction. The value of LTE depends on the type of base layer, reinforcement ratio, and crack stiffness. LTE is not a reliable indicator for predicting the punchout potential during regular construction regardless of a slab thickness and crack spacing higher than 90%, and with sufficient longitudinal reinforcement. Additionally, LTE values for a crack where a punchout process was performed remained at a relatively high level before and right after the development of the punchout [26].

The calculation for LTE adopted in the MEPDG is influenced by constant variables which are contributed by the base layer; the only parameter that changes over time and affects the LTE calculations is the variation in transverse crack stiffness (J_c,i_).

### 4.5. Deflection

**Figure 11:**
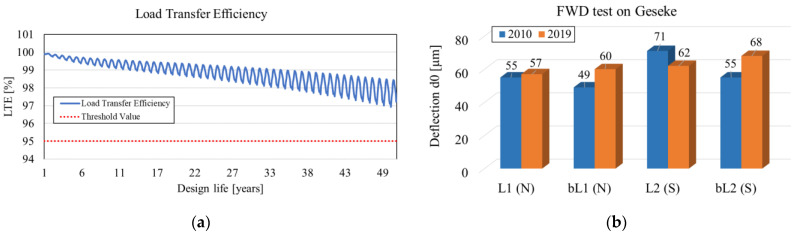
Different findings from design method and on-site measurements for Geseke private road: (**a**) variation of LTE prediction over time from the MEPDG; (**b**) investigation of bearing capacity (FWD) from 2009 to 2019.

An examination of the deflections was carried out using a falling weight deflectometer (FWD) in an interval of 20 m in the longitudinal direction [12]. The analysis of the FWD measurements on the Geseke private road in a timeframe of 10 years (Figure 11b) indicated that the FWD values on rolling lane 1 (N), between rolling lane l (N), and between rolling lane 2 (S) became larger. The bearing capacity was reduced, although, on the basis of the design procedures, a sufficient LTE is expected within the design life of 49 years (Figure 11a), and no punchout formation is expected within this period. The FWD was decreased only on rolling lane 2 (S), which can be justified by the influence of the traffic load in this area.

**Figure 12:**
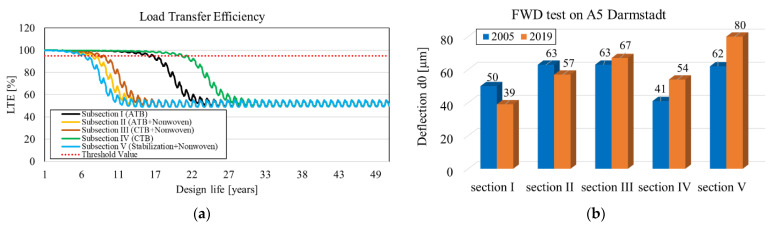
Different findings from design method and on-site measurements for A5 near Darmstadt: (**a**) variation of LTE prediction over time from the MEPDG; (**b**) investigation of bearing capacity (FWD) from 2005 to 2019.

The calculations of LTE according to the MEPDG for the A5 near Darmstadt also do not agree with FWD measurements in a timeframe of 15 years after construction of the CRCP. The evaluation of FWD measurements showed a reduction in bearing capacity on sections III, IV, and V of A5 near Darmstadt, although, according to the MEPDG, section IV consisting of cement-treated base should provide satisfactory LTE up to the 23rd year after installation. In the case of section II consisting of asphalt base course, the bearing capacity increased after 15 years, although, according to calculations, the LTE should fail after 7 years (Figure 12).

**Figure 13:**
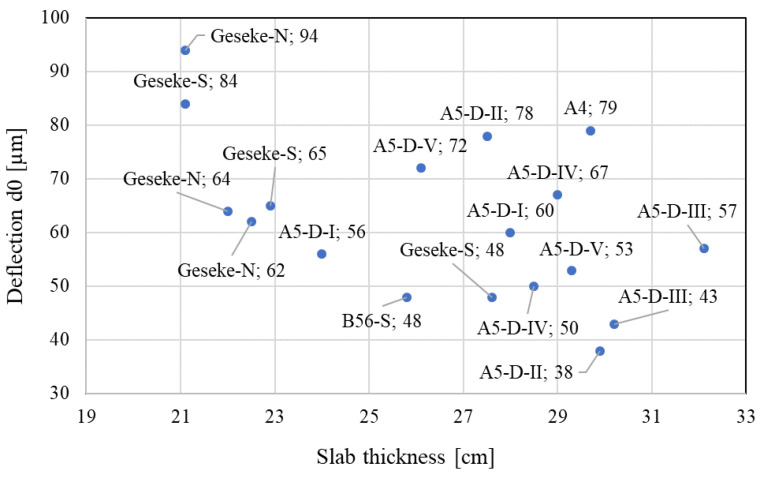
Average deflection in CRCP sections with different slab thickness.

Figure 13 summarizes the results of the FWD investigation on considered test sections with various slab thicknesses, base types, and pavement ages, and in different environmental regions depending on CRCP thickness and the overlayer on top of the concrete (if available). The base erodibility is calculated according to the MEPDG as follows:

(5) $$RE_{i}=\frac{\left(-0.37+0.0171P_{200}+0.0779\;EROD+0.0117\;PRECIP\right)}{12},$$

where *RE_i_* is the monthly rate of base erosion from the slab edge [in/month], *P*_200_, is the percentage subgrade passing the no. 200 sieve, *EROD* is the erodibility index depending on the material from 1 to 5, and *PRECIP* is the mean annual precipitation [in]. The erosion based on Equation (4) is completely dependent on properties of the base layer, and the influence of different shoulder types and heavy traffic volumes is not included. The effect of deflection in the calculations is not considered, although the deflection measurements on the investigated sections proved the erosions.

Figure 13 indicates that the mean deflections decrease with an increase in the concrete and top layer thickness. It appears that deflection and bearing capacity measurements are better and more reliable characteristics to predict the CRCP structural condition, as well as punchout prediction, compared to LTE.

The base layer is critical in terms of transmitting the stresses and maintaining the stability of the structure, and it influences the behavior of the CRCP on different aspects such as friction between slab and base layer or erosion. On one hand, a high friction will lead to higher stresses and a homogeneous crack spacing. On the other hand, a total bond between the pavement and the cement-treated base layer should be avoided as they will act as one, with a consequently reduced reinforcement rate. The placement of a geotextile between the CRCP and cement-treated base layer will accordingly act as a stress relief layer and allow both layers to deform separately. Although the implementation of geotextile on CTB resulted in a uniform crack pattern and erosion traces were not observed, this was not considered in the MEPDG design. Additionally, requirements for base courses are usually not answered within the scope of computational dimensioning, but enter into the procedures obliquely via input parameters such as the modulus of subgrade reaction.

## 5. Conclusions

Due to significant discrepancies between predicted MEPDG values (for punchout, LTE, crack spacing, and crack width) of the re-evaluated sections and measured values on the sites, in this study, the limitations of the MEPDG design process were identified, and the punchout and LTE predictions, as well as crack width models, for CRCP characterized by MEPDG design were evaluated. The formation of transverse cracks is completed during the shrinkage period, and crack formation is not finished directly after the placement of concrete. The calculation of the crack width can be improved by the consideration of the concrete shrinkage as a function of time. In this way, the influence of crack formation during the shrinkage process or after completion of the shrinkage period can be incorporated. Moreover, in the crack width calculations, the concrete area contributing to the crack width can be limited. The linear correlation between the crack spacing and the crack width needs to be adjusted. After the evaluation of the FWD values on the test tracks, it is recommended to consider the initial as-constructed and measurable values such as FWD in the calculations for the prediction of the punchout or LTE. Due to the very important function of erosion and resulting pumping in the deterioration of CRCP, it is advisable to include the influence of the base layer and the influence of different shoulder type and heavy traffic volume or the effect of deflection in the calculations. LTE is quite high during regular construction before and shortly after the development of punchouts; with sufficient longitudinal reinforcement, it is not a reliable indicator for predicting the punchout potential. The effect of reinforcement cover and the month of construction on the development of punchouts is not directly included in the design calculations, although this impact has already been confirmed. An important parameter contributing to the development of punchouts is the presence of horizontal cracks in the reinforcement layer, which, despite confirmation of their impact, have not yet been imported into the calculations.

## Figures and Tables

**Table 1:** Technical data of investigated tracks. AS: asphalt interlayer, L: lean concrete, S: cement stabilization, G: geotextile, AT: asphalt base course, CTB: cement-treated base.

Section	Subsection	Base Layer		CRCP Thickness [cm]		Steel Content [%]		Transverse Cracks (Service Life [Year])	Steel Covering [cm]
		Type	Thickness [cm]	Nominal	Actual	Nominal	Actual	Per 100 m	
E313	-	AS/L	5/25	25	25.8	0.76	0.73	81 (8)	9
A5 B	II	AS/S	30/0.5	24	28.1	0.75	0.64	35 (1)	12
	III	G/S	30/0.5	24	26.9	0.75	0.67	27 (1)	12
	IV	G/S	30/0.5	25.5	26.1	0.70	0.69	36 (4)	12
A5 D	I	AT	15	24	25.9	0.75	0.69	81 (4)	11
	II	AT/G	15/0.5	24	28.2	0.75	0.64	197 (4)	11
	III	CTB/G	15/0.5	24	26	0.75	0.69	217 (4)	11
	IV	CTB	15	24	25.7	0.75	0.70	216 (4)	11
	V	S	25	24	25.7	0.75	0.70	180 (4)	11
E34	-	AS/L	5/20	23	22.9	0.76	0.75	71 (8)	9
A94	-	AT	10	24.5	26	0.73	0.69	79 (9)	12.5
Geseke	-	AT	10	22	22.6	0.82	0.79	105 (11)	9
A4	-	L	20	23	24.4	0.76	0.72	77 (15)	10
B56	-	AT	15	22	21.1	0.61	0.64	125 (22)	9
E40	-	AS/L	6/20	20	23	0.85	0.75	-	9

## Data Availability

Not applicable.
